# RCC2 promotes breast cancer progression through regulation of Wnt signaling and inducing EMT

**DOI:** 10.7150/jca.36430

**Published:** 2019-11-01

**Authors:** Zhen Chen, Wenjing Wu, Yongsheng Huang, Limin Xie, Yu Li, Hengxing Chen, Wenjia Li, Dong Yin, Kaishun Hu

**Affiliations:** 1 Guangdong Provincial Key Laboratory of Malignant Tumor Epigenetics and Gene Regulation, Medical Research Center, Sun Yat-Sen Memorial Hospital, Sun Yat-Sen University, Guangzhou, 510120, China; 2 Department of Breast Oncology, Sun Yat-Sen Memorial Hospital, Sun Yat-Sen University, Guangzhou, 510120, China

**Keywords:** Regulator of Chromosome Condensation 2 (RCC2), Breast cancer, Epithelial-Mesenchymal Transition (EMT), Wnt signaling pathway

## Abstract

Regulator of chromosome condensation 2 (RCC2), also known as TD-60, is an RCC1 family member and plays an essential role in mitosis. However, the roles of RCC2 in breast cancer are still unclear. In this study, RCC2 was found to exert oncogenic activities in breast cancer. Samples of breast cancer tissue revealed an increased level of RCC2 and a high level of RCC2 was associated with poor overall survival rate of breast cancer patients. Overexpression of RCC2 significantly enhanced cell proliferation and migration abilities of breast cancer cells *in vitro* and *in vivo*. Mechanistically, RCC2 induced epithelial-mesenchymal transition (EMT) through the activation of Wnt signaling pathway. Collectively, our study indicates that RCC2 contributes to breast cancer progression and functions as an important regulator of EMT through the activation of Wnt signaling.

## Introduction

Breast cancer is the most common malignancy diagnosed and the leading cause of cancer death among females worldwide, accounting for 24.2% of cancer cases and 15% of cancer-related death 1-3. Many effective modalities have been utilized in the past few decades to successfully reduce cancer mortality rate. However, elucidating its biology and pathogenesis will allow far more informed diagnostic and therapeutic decisions needed by cancer patients.

Regulator of Chromosome Condensation 2 (RCC2) was originally identified as a candidate passenger protein 4. The passenger proteins are essential to facilitation of proper chromosome segregation and cell cleavage 5, and are defined by their redistribution during the mitosis process, from the inner centromeres in early mitosis to the spindle midzone and midbody upon mitotic exit 6. RCC2 expression was specific to late G2 and mitosis, and its localization corresponded precisely with the chromosomal passenger complex (CPC) 7. The process that RCC2 bound and activated kinase Aurora B, the catalytic subunit of CPC, was required for the recruitment of CPC to centromere and the following proper spindle formation and function 8.

Sequence analysis reveals that RCC2 is a member of the RCC1 family of guanine nucleotide exchange factors (GEFs) 9. Accumulating evidence showed that RCC2 exhibited GEF activity and was important to proper cell cycle progression both in interphase and mitosis. Its preference for binding with the nucleotide-free of the small GTPase Rac1 indicated that RCC2 was a potential exchange factor 9. Papini* et al.* discovered that RCC2 was a bona fide GEF for RalA, influencing the localization and activity of the CPC at centromeres during early mitosis 7. The absence of RCC2 expression arrested cells in prometaphase and activated the spindle assembly checkpoint 9. Interactome assays demonstrated that RCC2 was a key component in fibronectin-dependent adhesion signaling pathways during interphase 10. Its vast association with cell cycle progression and cell signaling leads us to speculate that RCC2 is a key nodal protein that integrates cell proliferation and cellular migration.

In this study, using publicly available online data and clinical specimen-based analyses, we identified that RCC2 expression increased in breast cancer tissues and elevated RCC2 expression was associated with poor prognosis in breast patients.* In vitro* and *in vivo* experiments demonstrated that RCC2 promoted the growth, migration, and tumorigenicity of breast cancer through the activation of Wnt signaling pathway and inducing EMT. Our study highlights a novel role and a new regulatory mechanism of RCC2 in breast cancer progression.

## Materials and Methods

### Cell culture

MCF10A cells were cultured in DMEM/F12 (Thermo Fisher Scientific, MA, USA) supplemented with 5% horse serum (Gibco, Carlsbad, USA), 20 ng/ ml EGF (Thermo), 0.5 mg/ ml hydrocortisone (Sigma, St. Louis, USA), 100 ng/ ml cholera toxin(Sigma), 10 mg/ml insulin (Gibco) and Penicillin/Streptomycin (Gibco). 293T cells and human breast cancer cell lines MDA-MB-468, JIMT1, MDA-MB-231 were cultured in Dulbecco's Modified Eagle's Medium (DMEM) supplemented with 10% fetal bovine serum (FBS, Invitrogen, CA, USA). MCF7, T47D and HCC1937 cells were cultured in RPMI-1640 (Gibco) with 10% FBS. All cell lines were incubated in a humidified incubator at 37 °C with 5% CO2.

### Lentivirus production and oligonucleotide transfection

Lentiviruses were produced by transfecting 293T cells with expression plasmids and packaging plasmids (psPAX2 and pMD2.G, Addgene_12260 and Addgene_12259); the supernatants were collected 48 hrs later, filtered through 0.45 mm filters (Millipore, CA, USA) and then concentrated via Amicon Ultra centrifugal filters (100KD MWCO, Millipore). Cells were transfected with the lentiviral particles in the presence of 8-μg/ mL polybrene (Sigma). Two days after infection, puromycin (1-μg/ mL) was added for 48-72 hrs to eliminate uninfected cells. siRNAs (GeneChem, Suzhou, China) were transfected using Lipofectamine RNAiMAX (Invitrogen). According to knockdown effects, siRCC2-2 and siRCC2-3 were used in our study. The sequence of siRCC2-2 was 5′- AAGGGGCAGCTGGGACATGGT -3′. The sequence of siRCC2-3 was 5′- GCUGUUAAAGAGGUCCAAATT -3′. Additionally, control siRNA (scramble) was also used in this study. The sequence of short hairpin RNAs (shRNAs) targeting human RCC2 (shRCC2) was 5′- AAGAGATGAAAGTGAGACTGA -3′.

### Immunoblotting

The collected tissues and cultured cells were lysed in RIPA lysis buffer (150 mM NaCl, 0.5% EDTA, 50 mM Tris-HCL, pH 8.0, 0.5% Nonidet P40) supplemented with protease inhibitors and phosphatase inhibitors (Roche, Mannheim, Germany) and centrifuged for 20 min at 14,000 x g and 4°C. Protein concentration was determined by bicinchoninic acid (BCA) assay (Cwbiotech, Beijing, China). Protein lysates were resolved by SDS-polyacrylamide gel electrophoresis, transferred to a PVDF membrane (Merck Millipore, CA, USA), and incubated with the indicated primary antibodies coupled with HRP-conjugated secondary antibodies by ECL reagent (Beyotime, Shanghai, China). Antibodies used were as follows: RCC2, E-cadherin, ZO1, N-cadherin, ZEB1, Snail1 (Cell Signaling Technology, Massachusetts, USA); FN1 (Abcam, Massachusetts, USA); β-catenin, c-Myc (Santa Cruz, TX, USA); Cyclin D1 (BD Biosciences, CA, USA). Secondary antibodies used were: HRP-goat anti-mouse, HRP-goat anti-rabbit (TransGene, Beijing, China).

### Quantitative real-time PCR (qPCR)

Total RNA was extracted using TRIzol (Thermo Fisher Scientific, MA, USA) and was transcribed into cDNA using PrimeScript RT Master Mix (Takara, Dalian, China) according to the manufacturer's instructions. qPCR was performed using the LightCycler ® 480 SYBR Green I Master (Roche) on a CFX96TM Real-Time System (BIO-RAD, California, USA). The relative gene expression levels were calculated using the ΔCt method (Ct of GAPDH minus the Ct of the target genes). Each experiment was performed in triplicate. Primer sequences are listed in Supplementary Table S1.

### Cell growth and colony formation assay

Cell growth was evaluated by MTT assay. Briefly, Cells were seeded in 96-well plates (1000 cells/ well) in triplicate and cell viability was examined by MTT dye solution (5 mg/ ml, Sigma) every two days. For colony formation assay, cells were seeded in 6-well plates (800cells/ well) in triplicate and cultured under normal growth conditions for two weeks. Colonies were washed and stained with 0.1% crystal violet, and were counted using an inverted microscope.

### Transwell Assay

Transwell assay was performed in 24-well plates (Corning, MA, USA) to assess cell migration. The cells were suspended in serum-free medium and incubated in the top chamber, and medium containing 10% FBS was placed as a chemical attractant in the bottom of the chamber. After incubation for 24 hrs, the cells attached to the membrane in the upper chamber were removed using a cotton swab, and the remaining cells were fixed with 4% paraformaldehyde (PFA) for 20 min. The migrated cells were stained with 0.1% crystal violet and observed via optical microscopy.

### Animal studies

All animal experiments were performed according to the ethical standards and national guidelines and were approved by the Animal Ethical and Welfare Committee (AEWC). Female BALB/c nude mice (4-5 weeks old) were purchased from Guangdong Medical Experiments Animal Center. For subcutaneous inoculation, 1×10^6^ cells in 200 μl PBS were injected subcutaneously into the right dorsal flank of 6-week-old female nude mice. Tumor volumes were measured with calipers every 3 days using the formula (length×width^2^)/2. For metastasis assay, 1×10^6^ cells in 200 μl PBS were injected into tail veins of 6-week-old female nude mice. After 6 weeks the mice were sacrificed, and the tumor nodules formed in the liver and lung were counted and then embedded in paraffin for hematoxylin and eosin (HE) staining.

### Immunofluorescence

Cells seeded in confocal dishes were fixed with 4% PFA for 20 min, and permeabilized with 0.5% Triton X-100 for 20 min. Following PBS washings, non-specific antigen binding sites were blocked by 2% Bovine Serum Albumin (BSA) for 30 min. Cells were then incubated with anti-β-catenin (Santa Cruz, 1:100) antibodies overnight at 4 °C. After washing with PBS, cells were incubated with secondary antibody (DyLight 488-conjugated mouse anti-rabbit IgG; 1:200) for 60 min and the nuclei were stained with DAPI (Invitrogen) for 5 min, which was subsequently washed with PBS. All experiments were light-sensitive. The cells were then viewed with a fluorescence microscope (Olympus Corporation, Tokyo, Japan).

### Statistical analysis

Statistical analyses were performed using SPSS 20.0 software (IBM Corporation, Armonk, NY, USA) and GraphPad Prism6 software (La Jolla, CA, USA). The data are presented as the mean ± standard deviation and all experiments were done in triplicates. Mann-Whitney U-test was used to assess differences in the RCC2 mRNA expression levels in tumorous and normal tissues. The statistical comparisons were analyzed using Student's t-test (only two groups) or ANOVA (three or four groups). Survival curves were obtained using the Kaplan-Meier method, and the log-rank test was used to test the difference in survival curves.* P* < 0.05 (two-sided) was considered statistically significant.

## Results

### High RCC2 expression in breast cancer correlates with survival of patients

**Figure 1:**
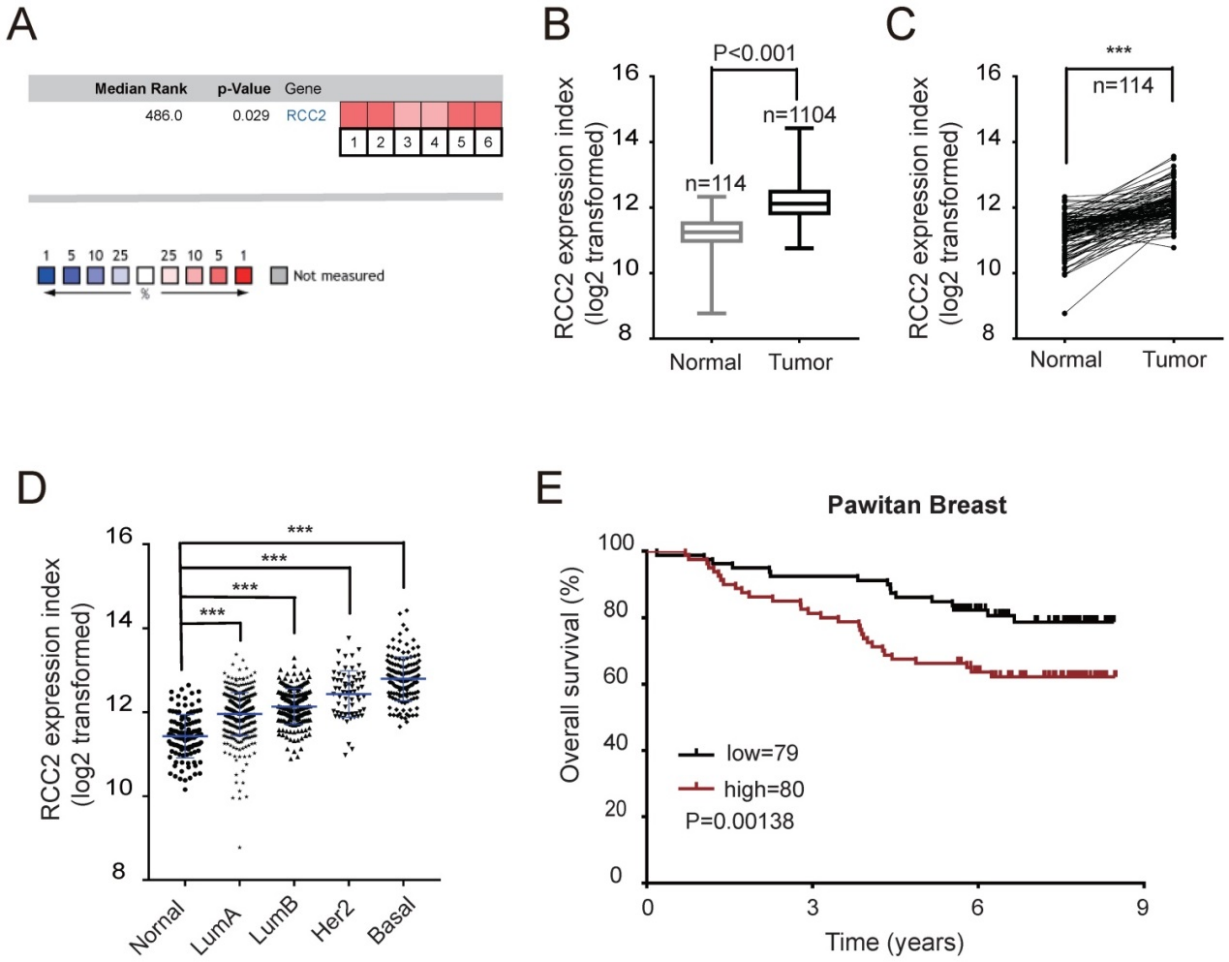
** Up-regulation of RCC2 is associated with poor prognosis of breast cancer patients. (A)** A meta-analysis of 6 independent breast cancer datasets in Oncomine database. **(B-D)** RCC2 mRNA expression from TCGA database was compared between 114 normal breast tissues and 1104 breast cancer tissues(B), between 114 pairs of breast cancer tissues with their adjacent normal breast tissues(C), and across four different subtypes in breast cancer(D). **(E)** Kaplan-Meier curves indicating the overall survival based on the expression levels of RCC2 in breast cancer patients derived from the publicly accessible Pawitan Breast Dataset (log-rank test, *P*=0.0138). ***P < 0.001.

To elucidate the potential relationship between RCC2 expression and cancer incidence, RCC2 expression level was examined via Oncomine Online Database (https://www.oncomine.org/resource/main.html) 11. Gene Summary analyses showed that RCC2 had significantly higher expression in several cancers, including lymphoma, breast, cervical, colorectal, gastric, liver, lung, and ovarian cancer (*p* value, 1E-4; fold change, 2; gene rank, top 10%, **Figure S1**). Herein, we focused on breast cancer and selected six independent datasets for a meta-analysis, and found that RCC2 mRNA expression was significantly up-regulated in breast cancer tissues compared with normal counterparts (**Figure 1A**, *P*=0.029). To further evaluate the reliability of this observation as obtained in Oncomine, RCC2 mRNA expression was next analyzed in 1104 breast cancer tissue samples and 114 normal breast tissue samples from The Cancer Genome Atlas (TCGA) Data Portal (https://cancergenome.nih.gov/). The results indicated that RCC2 was up-regulated in breast cancer tissues (**Figure 1B**, *P* < 0.001). Meanwhile, a comparison of RCC2 expression between 114 pairs of breast cancer tissues with their adjacent normal breast tissues validated the trend above (**Figure 1C,** *P* < 0.001).

Breast cancer is clinically divided into four major molecular subtypes based on the expression of the estrogen receptor (ER), progesterone receptor (PR), and human epidermal growth factor receptor 2 (HER2), including luminal A, luminal B, Her2, and basal-like sub-types 12. Interestingly, the highest RCC2 expression was found in the basal-like breast cancer (**Figure 1D**), which is known to have a propensity for metastasis and worse prognosis 13. Kaplan-Meier survival analysis of Pawitan cohort 14 revealed that patients with high RCC2 expression had poorer overall survival on the Oncomine database (**Figure 1E**). Taken together, these results indicate that RCC2 is up-regulated in human breast cancer tissues, which may play a significant role in the development of breast cancer and the clinical prognosis of breast cancer patients.

### RCC2 mediates oncogenic activities in breast cancer cell *in vitro*

**Figure 2:**
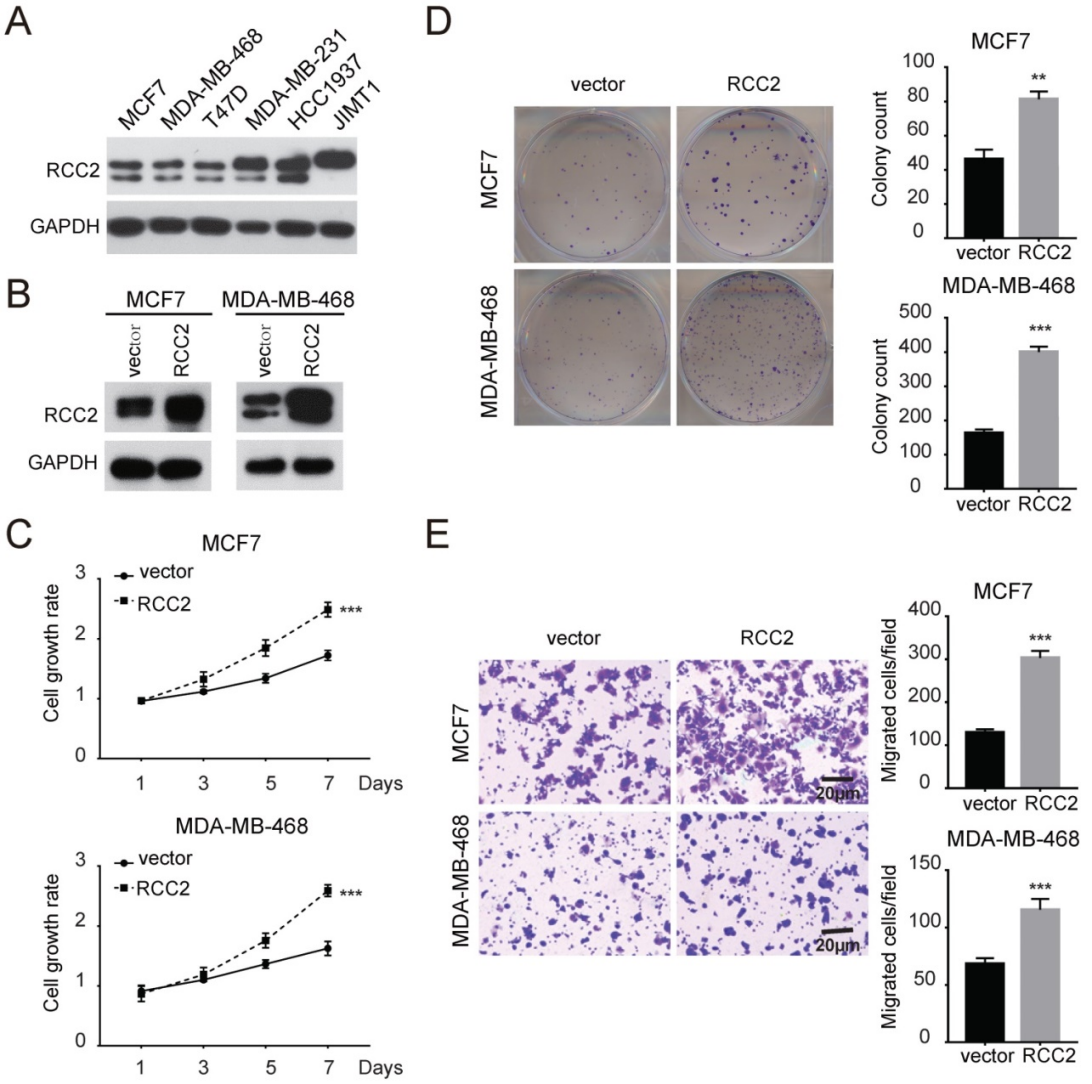
** Elevated RCC2 expression accelerates breast cancer cell proliferation and migration *in vitro.* (A)** The protein levels of RCC2 in human breast cancer cell lines were determined by western blotting. **(B)** RCC2 transfection efficiencies in MCF7 and MDA-MB-468 cells were confirmed. Proliferation capability of breast cancer cells transfected with RCC2 overexpression and control vector was evaluated by MTT assays **(C)** and Colony formation assays** (D)**.** (E)** Transwell assays showed that RCC2 overexpression promoted cell migration. Each bar represents mean ± SD of three independent experiments. **P < 0.01; ***P < 0.001.

**Figure 3:**
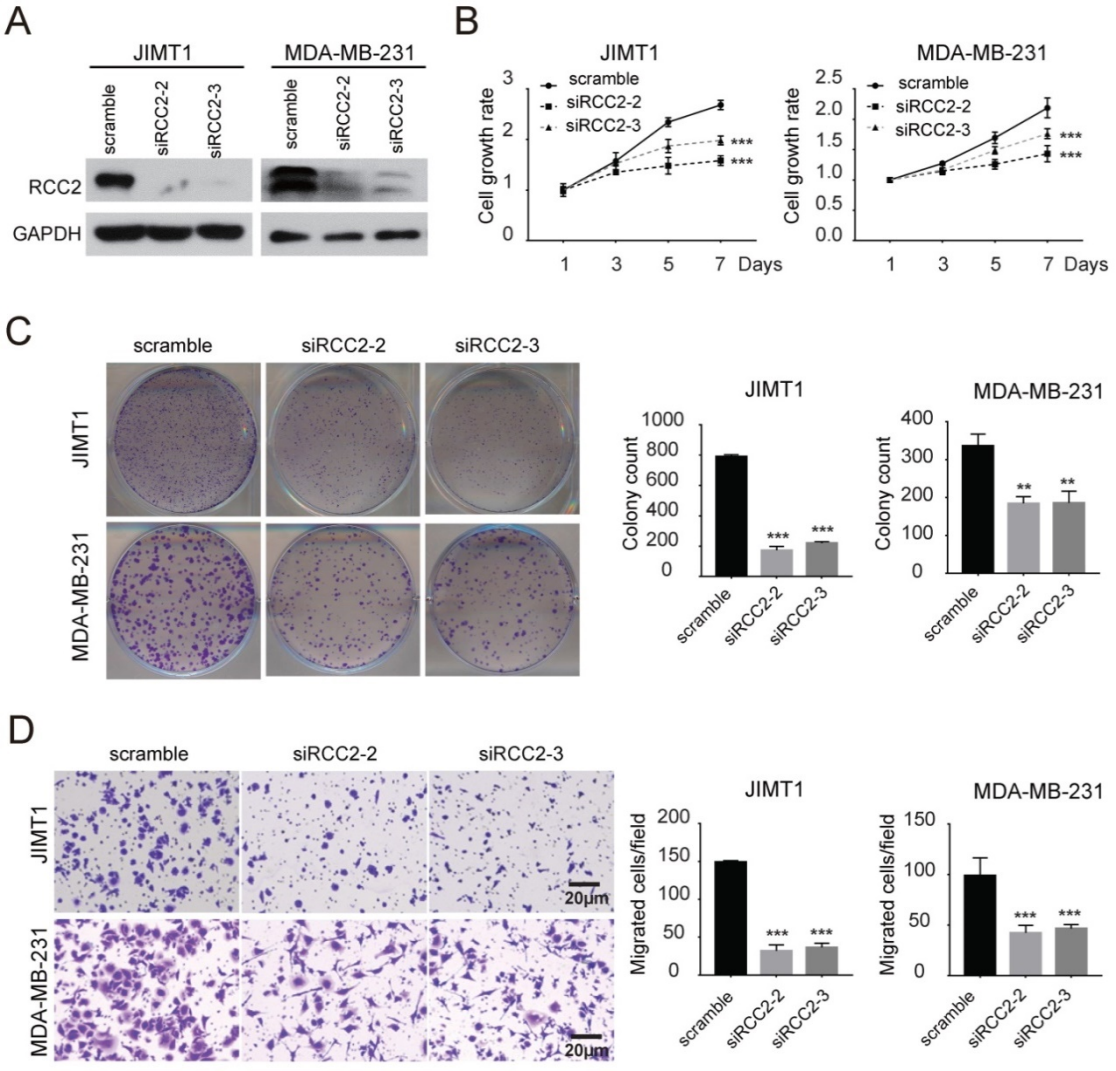
** RCC2 knockdown attenuates the proliferation and migration of breast cancer cell *in vitro.* (A)** Silencing of RCC2 in JIMT1 and MDA-MB-231 cells, transfected with non-targeting siRNA (scramble) or two different RCC2-specific siRNA, attenuated its expression at protein level. RCC2 knockdown suppressed breast cancer cell growth and cell migration, evaluated by MTT assays **(B)**, colony formation assays **(C)**, and Transwell assays **(D)**. Results are expressed as mean ± SD of three independent experiments. **P < 0.01; ***P < 0.001.

Next, we investigated the role of RCC2 on malignant phenotypes of breast cancer cells. The expression levels of RCC2 in breast cancer cell lines were then measured (**Figure 2A**). We increased RCC2 expression in MCF7 and MDA-MB-468 cells via lentiviral infection and depleted RCC2 in JIMT1 and MDA-MB-231 cells using siRNA. Cell growth assays revealed that stable RCC2 overexpression (**Figure 2B**) accelerated cell proliferation as determined by MTT (**Figure 2C**) and colony growth assays (**Figure 2D**). Additionally, exogenous RCC2 expression in breast cancer cells dramatically enhanced the cell migratory capability as indicated by Transwell assays (**Figure 2E**). In contrast, knockdown of RCC2 in JIMT1 and MDA-MB-231 cells (**Figure 3A**) attenuated cell viability (**Figure 3B**), clonogenicity (**Figure 3C**) and migratory properties (**Figure 3D**). Taken together, our results substantiate RCC2 as an oncogene promoting the proliferation and migration of breast cancer cells.

### Silencing of RCC2 decreases xenograft tumor growth and metastasis *in vivo*

**Figure 4:**
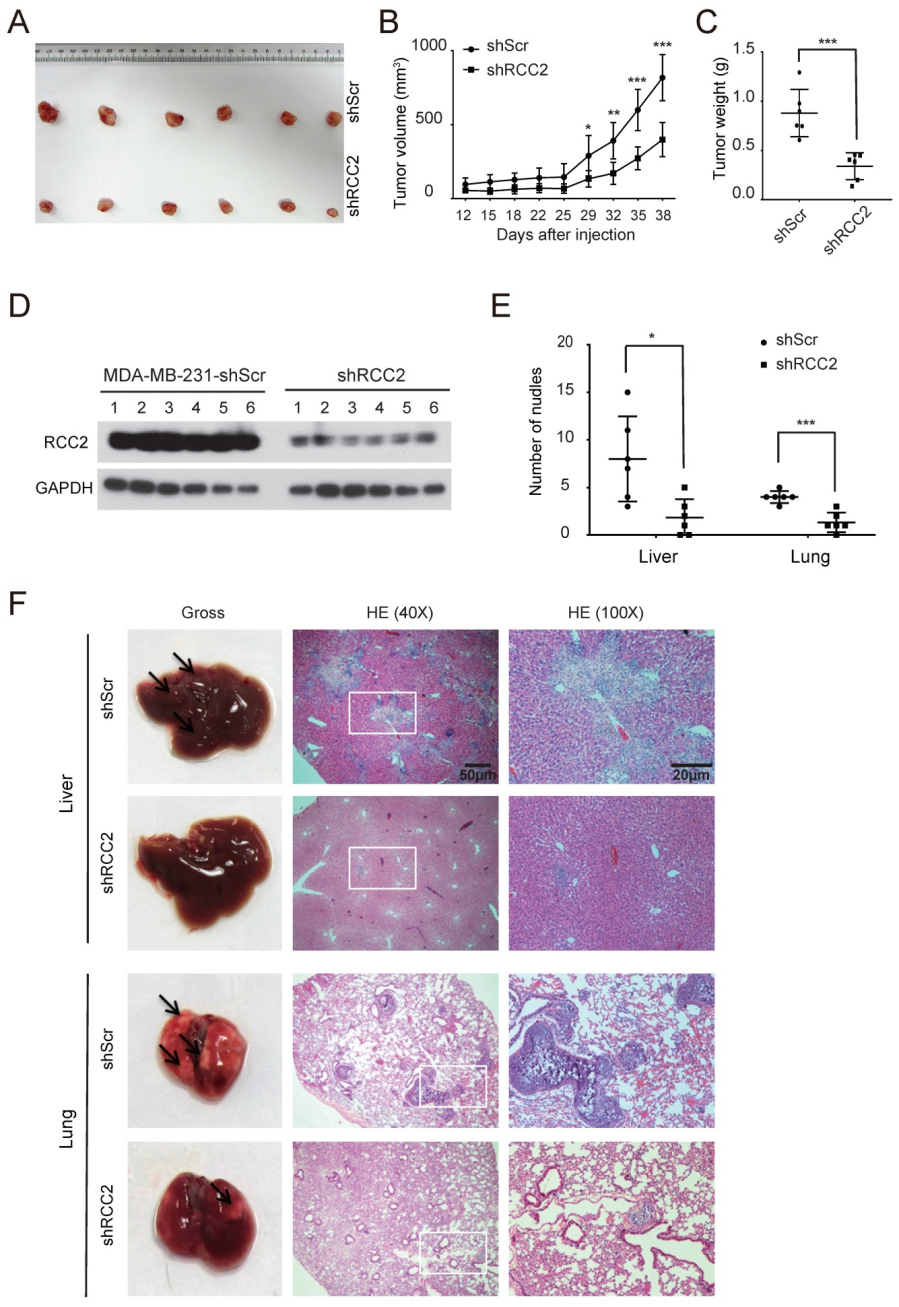
** Depletion of RCC2 diminishes xenograft tumor growth and metastatic potential* in vivo.* (A-C)** MDA-MB-231 with RCC2 stably knock-downed and control cells were injected into 6-week-old BALB/c female nude mice (n=6). After 6 weeks of injected, xenograft tumors were harvested. Photographs of harvested tumors (A), tumor growth curves (B), and tumor weight (C) were shown. **(D)** RCC2 protein levels in a murine subcutaneous xenograft model, as confirmed by western blotting. **(E)** Quantitative analyses of metastases were measured in liver and lung tissues. **(F)** Liver and lung metastases of MDA-MB-231 cells were determined by tissue observation and HE staining. *P < 0.05, **P < 0.01, ***P < 0.001.

To investigate the effects of RCC2 on the tumorigenic capacity of breast cancer cells *in vivo*, xenograft tumor growth assay was established by subcutaneous transplantation with either MDA-MB-231-shRCC2 or MDA-MB-231-shScr cells (n=6) (**Figure 4A**). Consistent with *in vitro* results, silencing of RCC2 markedly reduced tumor size and weight as compared to the scramble group (**Figure 4B** and **4C**). Western blotting confirmed lower expression of RCC2 in MDA-MB-231-shRCC2 xenograft tumors (**Figure 4D**). Furthermore, to delineate whether RCC2 could promote tumor metastasis *in vivo*, MDA-MB-231-shRCC2 or negative control cells were intravenously injected into nude mice (n=6) via tail vein to establish a liver/lung metastatic model. At the completion of experiment, livers and lungs were resected. The visible tumor metastases were statistically and numerically lower in MDA-MB-231-shRCC2 group (**Figure 4E**). Metastatic nodules on the surfaces of livers and lungs were further confirmed by hematoxylin-eosin (HE) staining (**Figure 4F**). In summary, these results suggest that silencing of RCC2 significantly attenuates xenograft tumor progression and metastatic potential *in vivo.*

### RCC2 induces EMT via activating Wnt/β-catenin signaling pathway

**Figure 5:**
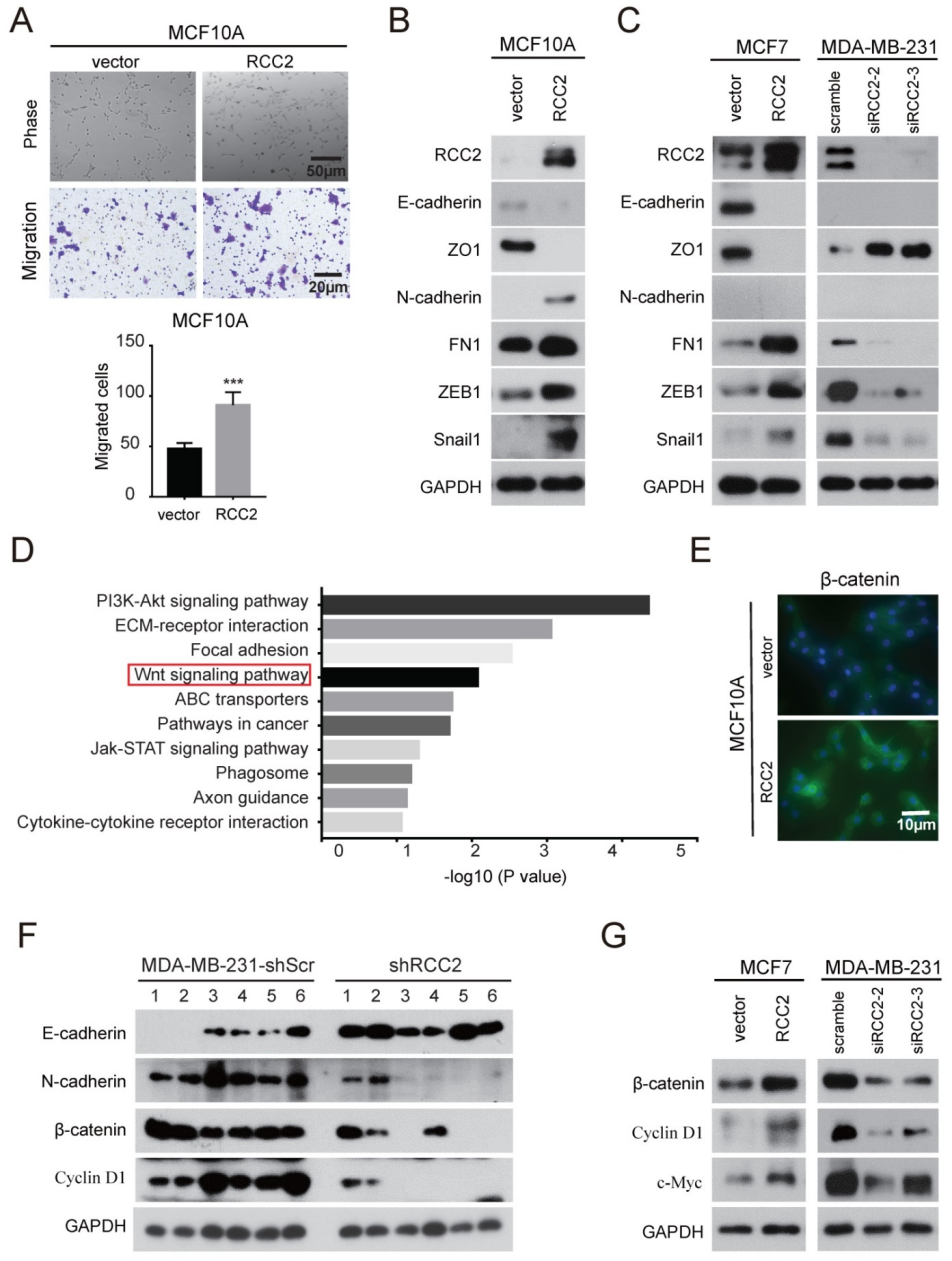
** RCC2 was correlated with EMT and Wnt signaling pathway in breast cancer cells. (A)** The morphology of MCF10A cells stably expressed RCC2 and control by bright field microscopy (upper). Representative images show that RCC2 overexpression promoted MCF10A cells migration (down). Immunoblotting analysis of EMT markers in MCF10A** (B)** and MCF7 cells (**C,** left) with RCC2 overexpressed, or MDA-MB-231 cells with RCC2 down-regulated (**C,** right). **(D)** The top 10 categories of the KEGG pathways. KEGG enrichment was performed based on differentially expressed mRNAs by defining a threshold of the average FPKM ≥ 1, and the cut-off as more than a 2-fold change in MDA-MB-231 cells transfected with scramble or two RCC2-targeting siRNAs. **(E)** Immunofluorescence staining for β-catenin in MCF10A cells after transduction with exogenous RCC2 and control. **(F)** Expression of EMT markers (E-cadherin, N-cadherin) and the Wnt pathway downstream molecules (β-catenin, Cyclin D1) in xenograft models from MDA-MB-231-shScr and shRCC2 groups were detected by western blotting. **(G)** Western blotting analysis of Wnt target genes expression in response to RCC2 overexpression or knockdown in MCF7 and MDA-MB-231 cells. ***P < 0.001.

Accumulating evidence suggests that EMT, a process by which epithelial cells acquire the characteristics of mesenchymal cells, plays a critical role in breast cancer 15. In order to investigate whether RCC2 regulates EMT, we stably expressed RCC2 in MCF10A cell, a "normal" nontumorigenic breast epithelial cell line that had been extensively used to study EMT 16. As shown in **Figure 5A**, exogenous RCC2-tranfected cells rendered a mesenchymal morphology and acquired migratory capability. Epithelial markers like E-cadherin and ZO1 were down-regulated, whereas mesenchymal markers N-cadherin and fibronectin were significantly up-regulated. Transcription factors such as ZEB1 and Snail1, whose high expression were considered a hallmark of EMT through transcriptional control of E-cadherin 17, were also showed an up-regulation in MCF10A cells with RCC2 overexpression (**Figure 5B**). Consistently, overexpression of RCC2 promoted EMT in epithelial-like MCF-7 cells, while knockdown of RCC2 diminished EMT progression in MDA-MB-231 cells as EMT markers indicated (**Figure 5C**).

To probe the underlying mechanisms of RCC2 function in breast cancer, whole genome transcriptome analysis using RNA-seq was performed on MDA-MB-231 cells transfected with control or RCC2-targeting siRNAs. Two independent siRNAs produced highly similar results (**Figure S2**). Kyoto Encyclopedia of Genes and Genomes (KEGG) analysis of differential expression genes indicated that RCC2 loss-of-function affected pathways enriched mainly in PI3K-AKT signaling, ECM-receptor interaction, focal adhesion, Wnt signaling, etc (**Figure 5D**). Wnt signaling pathway is reported to coordinate EMT programs 18. Thus, to elucidate whether the Wnt pathway is involved in RCC2-induced breast cancer progression, expression of β-Catenin in MCF10A cells was examined by immunofluorescence. **Figure 5E** showed that MCF10A cells stably expressing RCC2 resulted in an increased β-Catenin level. To confirm that the increased expression of β-catenin was due to activated Wnt signaling pathway, we further detected the levels of Wnt signaling downstream molecules in xenograft model. As indicated in **Figure 5F**, expression of EMT markers, β-catenin and Cyclin D1 were significantly decreased in MDA-MB-231-shRCC2 xenograft tumors. Moreover, overexpression of RCC2 in MCF-7 cells significantly increased, while knockdown of RCC2 in MDA-MB-231 cells decreased the expression of Wnt signaling genes, such as β-catenin, Cyclin D1 and c-Myc (**Figure 5G**). And their mRNA levels were further validated by quantitative real-time PCR (**Figure S3**). These data suggest that RCC2 have a pro-metastatic role in breast cancer, which is mediated via inducing EMT and activating Wnt-signaling pathway.

## Discussion

RCC2's roles in human cancers have been increasingly scrutinized in recent years. The dysregulation of RCC2 expression and its clinical significances have been documented. Elevated expression of RCC2 was correlated with poor prognosis in basal cell carcinoma (BCC) 19, colorectal cancer 20, gastric cancer 21, lung cancer 22-24 and ovarian cancer 23. Conversely in colorectal cancer, reduced RCC2 expression was associated not only with improved survival in microsatellite instable (MSI) patients, but also with poor prognosis in microsatellite stable (MSS) group 20. However, there is no study on elucidating the correlation between RCC2 and breast cancer progression. Herein, we found RCC2 expression was up-regulated in several kinds of tumors via Oncomine database, including breast cancer. TCGA RNA-seq expression data further confirmed the higher RCC2 expression in cancer as compared to normal tissues. Kaplan Meier survival analysis of breast cancer patients showed that high expression of RCC2 had significantly reduced overall survival rates.

RCC2 is linked to CPC complexes involved in mitotic spindle assembly 7, membrane dynamics 25 and cell cycle progression 9. In addition, suppression of RCC2 blocks cellular activity in prometaphase 9, alters cell morphology 25, and increases apoptosis 23. Those evidence suggest the potential function of RCC2 in regulating cell motility. Our studies demonstrated that ectopic expression of RCC2 in breast cancer cells increased cell proliferation *in vitro*, whereas the silencing of RCC2 led to opposite phenotypes. In addition, our *in vivo* experiments showed subcutaneous injection of MDA-MB-231 cells with knock-downed RCC2 significantly inhibited tumorigenicity.

Recently, RCC2 has been identified as a regulator of cellular migration and tumor metastasis. Further study revealed that RCC2 interacted physically with the small GTPase RAC1 and may act as a GEF for Rac1 9. Another research implicated that RCC2, as a negative dual regulator of RAC1 and ARF6, guided mesenchymal cell directional migration 10. Williamson *et al*. identified that interactions among CORO1C, RCC2, and RAC1 accelerated mesenchymal cell migration through RAC1 trafficking and controlling its exposure to GEFs 25. CORO1C has been reported to be up-regulated in multiple clinically aggressive cancers and its down-regulation resulted in reduced cell invasion and metastasis 26, 27. Like before, transwell assays also demonstrated that exogenous RCC2 in breast cancer cells increased migration potential, while ablation of RCC2 markedly reduced their migration capacity. Metastatic viability was further assessed in tail vein injection mouse models. MDA-MB-231-shRCC2 injected mice showed a reduction in the number of metastatic nodules in liver and lung.

EMT is a prerequisite physiological process for metastasis in most cancers and several studies have suggested the association of RCC2 and EMT in cancer malignancy. RCC2 expression was elevated in lung adenocarcinoma (LUAD), up-regulation of RCC2 could promote cell migration and invasion through the activation of EMT 22. In another study, lncRNA *LCPAT1* was found to be involved in cigarette smoke extract (CSE) /PM2.5-induced lung cancer cell autophagy and EMT via RCC2 up-regulation 24. Here, we found that RCC2 overexpression promoted mesenchymal phenotypes in mammary epithelial MCF10A cells and western blotting results revealed that RCC2 stimulated EMT in breast cancer cells.

To elucidate the role of RCC2 in EMT, RNA-seq was performed in MDA-MB-231 cells with control or RCC2 silenced. Previous research on LUAD indicated that RCC2 might activate EMT through the activation of the MAPK-JNK signaling pathway 22. Our KEGG pathway analysis suggested that RCC2 loss-of-function affected the PI3K/AKT signaling, an upstream pathway of MAPK-JNK. While no significant difference was observed on the PI3K-AKT signaling pathway in breast cancer cells (data were not shown), another high-scored pathway, Wnt signaling pathway, was further investigated.

Wnt pathway is a key signaling cascade that regulates physiological germination and development of breast cancer 28, 29*.* Canonical Wnt signaling is also referred as Wnt/β-catenin signaling, defined by cytoplasmic β-catenin nuclear translocation and activation of TCF4/β-catenin transcriptional targets 30, 31. Wnt driven breast cancers are noteworthy as they illustrate increased cell motility, EMT phenotype and tumor metastasis 32-36. Immunofluorescent staining showed that up-regulation of RCC2 could increase the expression and nuclear translocation of β-catenin in MCF10A cell. Furthermore, activation of TCF4/β-catenin transcriptional targets, such as c-Myc and CyclinD1, play significant roles in regulating tumor malignancy and EMT 37, 38. Our results indicated that regulation of RCC2 level could directly affect the expression of β-catenin and its transcriptional targets.

Consequently, we generated RCC2-enriched and silenced cells to perform* in vitro* and *in vivo* assays, and found that RCC2 overexpression promoted aggressive progression of breast cancer through the activation of the Wnt signaling pathway. However, further investigations are needed to clarify deeper mechanisms that elaborate the roles of RCC2 in the regulation of Wnt signaling pathway.

## Supplementary Material

Supplementary figures and tables.

## Abbreviations

RCC2: Regulator of chromosome condensation 2
EMT: Epithelial-Mesenchymal Transition
CPC: chromosomal passenger complex
GEFs: guanine nucleotide exchange factors
MTT: methyl-thaizolyl-tetrazolium
KEGG: Kyoto Encyclopedia of Genes and Genomes
