# Supplementary material for: RCC2 promotes breast cancer progression through regulation of Wnt signaling and inducing EMT
Source: J Cancer. 2019 Nov 1;10(27):6837–47. doi: 10.7150/jca.36430 (PMC6909956; doi:10.7150/jca.36430)
Supplement: Supplementary file 1 — Supplementary figures and tables. [file jcav10p6837s1.pdf]

Supplemental figures and tables

Analysis Type by Cancer	Cancer vs. Normal	
Bladder Cancer		
Brain and CNS Cancer		
Breast Cancer	4	1
Cervical Cancer	2	
Colorectal Cancer	2	
Esophageal Cancer		
Gastric Cancer	2	
Head and Neck Cancer	1	
Kidney Cancer		
Leukemia		
Liver Cancer	1	
Lung Cancer	1	
Lymphoma	3	
Melanoma		
Myeloma		
Other Cancer	6	
Ovarian Cancer	2	
Pancreatic Cancer		
Prostate Cancer		
Sarcoma		
Significant Unique Analyses	24	1
Total Unique Analyses	342	

Figure S1 Gene Summary analyses of RCC2 in Oncomine database. A comparison of RCC2 expression levels between cancer and normal tissues showed that RCC2 was highly expressed in breast cancer, cervical cancer, colorectal cancer, gastric cancer, ovarian cancer and so on.

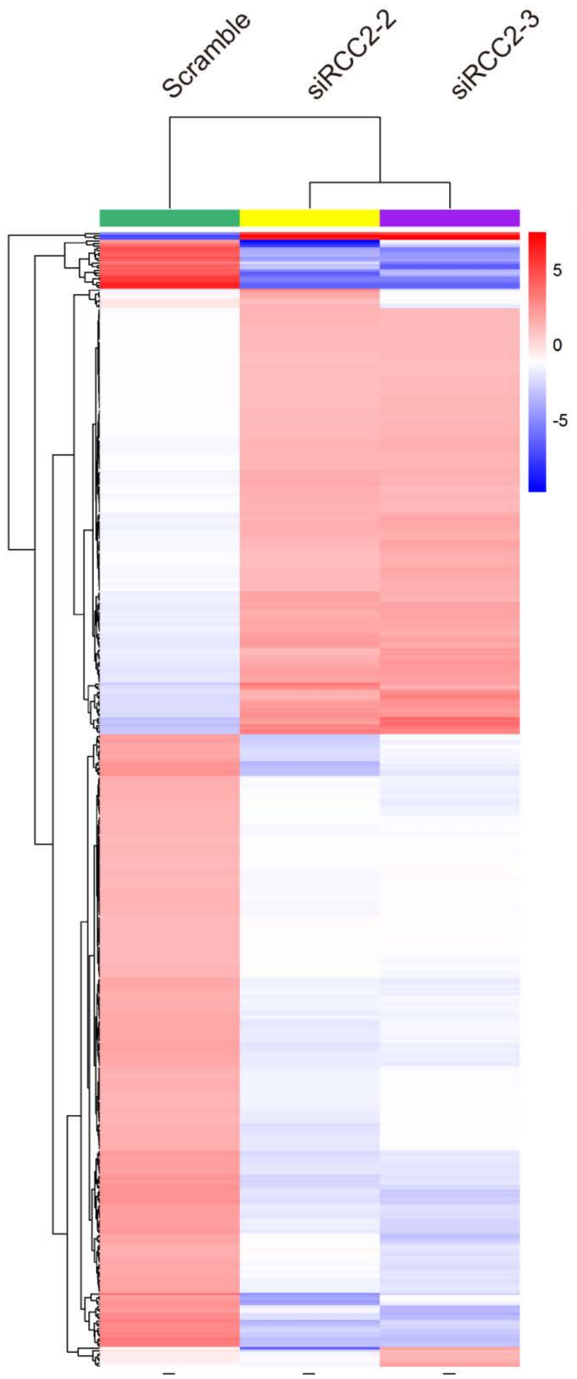

Figure S2 Heatmap of the RNA-seq results following RCC2 silencing.

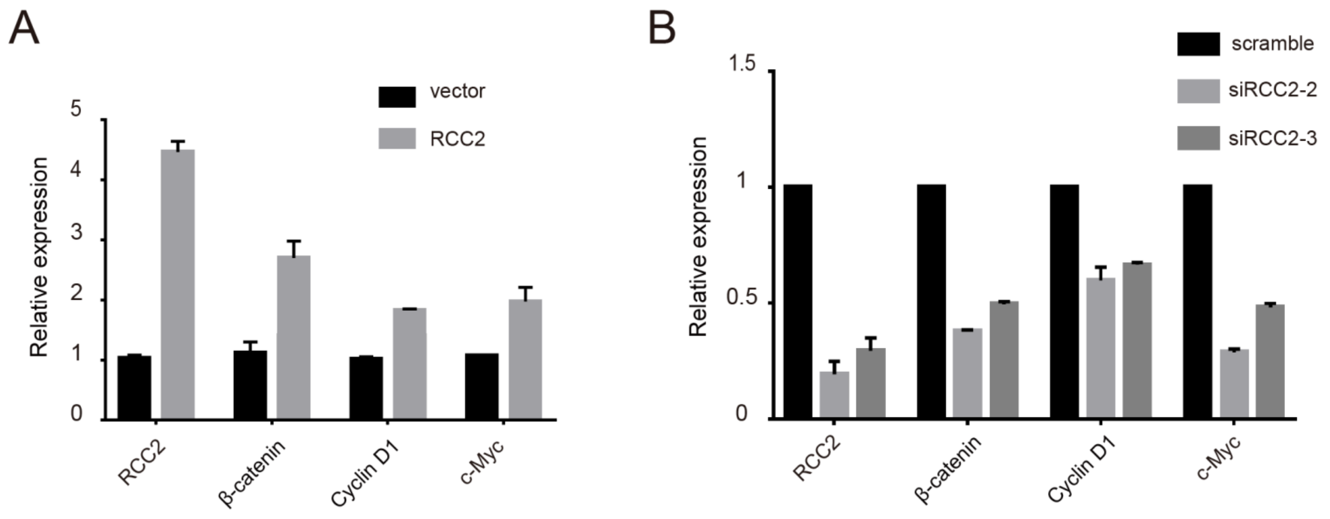

Figure S3 Real-time PCR analysis showing expression level of Wnt signaling genes in MCF7 cells with RCC2 overexpressed (A) or MDA-MB-231 cells with RCC2 silenced (B).

Supplementary Table S1 Sequences of primers used for Real-time quantitative PCR (5'--3').

Genes	Forward	Reverse
RCC2	TTTTCTCAGAGCAGGTCGCC	TTCGGGGGTTGTATTCTGGC
β-catenin	CTGAGGAGCAGCTTCAGTCC	CCATCAAATCAGCTTGAGTAGCC
Cyclin D1	ATCAAGTGTGACCCGGACTG	CTTGGGGTCCATGTTCTGCT
c-Myc	GGGTAGTGGAAAACCAGCCTC	AGAAATACGGCTGCACCGAG
GAPDH	GGTGGTCTCCTCTGACTTCAACA	GTTGCTGTAGCCAAATTCGTTGT
